# CardioGenBase: A Literature Based Multi-Omics Database for Major Cardiovascular Diseases

**DOI:** 10.1371/journal.pone.0143188

**Published:** 2015-12-01

**Authors:** Alexandar V, Pradeep G. Nayar, R. Murugesan, Beaulah Mary, Darshana P, Shiek S. S. J. Ahmed

**Affiliations:** 1 Faculty of Allied Health Sciences, Chettinad Academy of Research and Education, Kelambakkam 603 103, Tamil Nadu, India; 2 Department of Cardiology, Chettinad Super Specialty Hospital, Chettinad Academy of Research and Education, Kelambakkam 603 103, Tamil Nadu, India; 3 Department of Computational Biology, Drug discovery Lab, Faculty of Allied Health Sciences, Chettinad Academy of Research and Education, Kelambakkam, 603 103, Tamil Nadu, India; Harbin Medical University, CHINA

## Abstract

Cardiovascular diseases (CVDs) account for high morbidity and mortality worldwide. Both, genetic and epigenetic factors are involved in the enumeration of various cardiovascular diseases. In recent years, a vast amount of multi-omics data are accumulated in the field of cardiovascular research, yet the understanding of key mechanistic aspects of CVDs remain uncovered. Hence, a comprehensive online resource tool is required to comprehend previous research findings and to draw novel methodology for understanding disease pathophysiology. Here, we have developed a literature-based database, CardioGenBase, collecting gene-disease association from Pubmed and MEDLINE. The database covers major cardiovascular diseases such as cerebrovascular disease, coronary artery disease (CAD), hypertensive heart disease, inflammatory heart disease, ischemic heart disease and rheumatic heart disease. It contains ~1,500 cardiovascular disease genes from ~2,4000 research articles. For each gene, literature evidence, ontology, pathways, single nucleotide polymorphism, protein-protein interaction network, normal gene expression, protein expressions in various body fluids and tissues are provided. In addition, tools like gene-disease association finder and gene expression finder are made available for the users with figures, tables, maps and venn diagram to fit their needs. To our knowledge, CardioGenBase is the only database to provide gene-disease association for above mentioned major cardiovascular diseases in a single portal. CardioGenBase is a vital online resource to support genome-wide analysis, genetic, epigenetic and pharmacological studies.

## Introduction

Cardiovascular diseases are the leading cause of morbidity and mortality worldwide[1]. Among the cardiovascular conditions, cerebrovascular disease, coronary artery disease (CAD), hypertensive heart disease, inflammatory heart disease, ischemic heart disease and rheumatic heart disease are considered as major cardiovascular diseases (MCVDs) that are caused by both genetic and epigenetic factors resulting in heart failure. The pathophysiology of MCVDs are not merely the result of single gene defect or its product alone. It is an outcome of several molecules, which function collaboratively to initiate oxidative stress, inflammation, endothelial dysfunction and thrombosis. To date, the polygenic nature of MCVDs is highly accepted[2,3]. Several studies have been conducted on MCVDs which includes association studies, linkage studies and meta-analyses that identified various diseases-associated genes[4–9]. These findings generated an unprecedented amount of biological data that provide an opportunity to construct a useful gene resource for MCVDs.

A broad knowledge of genes and proteins involved in cardiovascular conditions is crucial for understanding of molecular mechanism in disease pathology. Here, we present a comprehensive gene database (CardioGenBase) for the major cardiovascular diseases. The CardioGenBase (http://www.CardioGenBase.com/) is a knowledge base which effectively integrates, analyzes and visualizes major cardiovascular disease associated research articles. It was constructed by collecting gene/protein information across MCVDs related published literatures. The identified entities were enriched with chromosomal location, gene ontology, gene expression, protein expression, bioavailability, pathways, SNPs, protein interaction network and drugs. In addition, it enables users to search and browse various data categories and data connections. CardioGenBase is a unique genetic resource that would help cardiovascular research community to design new experiments and to unveil novel disease mechanisms.

## Results and Discussion

**Fig 1:**
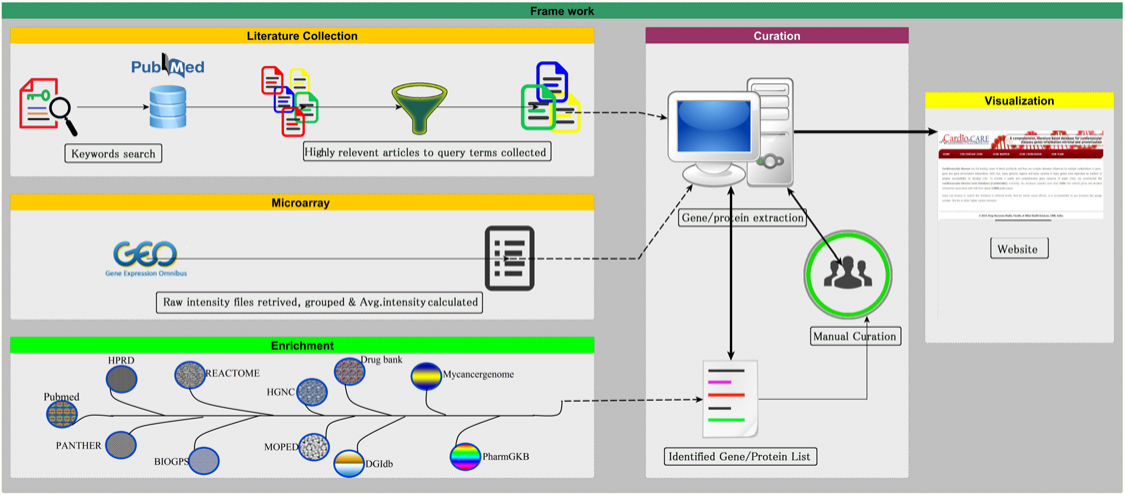
CardioGenBase Construction. The framework describes the construction of CardioGenBase. It includes data mining of biomolecules, filtration, curation, enrichment, system interface and visualization.

CardioGenbase was created as literature evidence based database to provide useful molecular information on major cardiovascular diseases (Fig 1). The scientific literature was manually collected, filtered and a computer program (Lucene)was used to identify gene/protein names from the collected articles. Lucene is an open source and a java based computer program. It is effective for full-featured text mining. Using this program, we identified 1365 genes for CAD, 240, 75, 28, 428 and 139 for cerebrovascular disease, hypertensive heart disease, inflammatory heart disease, ischemic heart disease and rheumatic heart disease, respectively (Table 1). The data obtained are categorized, stored and managed as tables using MySQL to create CardioGenbase.

**Table 1:** Text mining results. The number of literature collected for each cardiovascular disease. These literature was filtered based on title/abstract, relevance to the search terms to extract genes/proteins using a semi-automated method.

Disease	No. Literature	Genes Extracted
Cerebrovascular disease	1966	240
**Coronary Heart Disease**	17471	1365
**Hypertensive heart disease**	260	75
**Inflammatory heart disease**	23	28
**Ischemic heart disease**	5624	428
**Rheumatic heart disease**	644	139

The genes in the database were enriched with gene expression, protein expressions, ontology, SNP, PPI network, drugs and pathways. These molecular information is a prerequisite to design and conduct basic research to understand disease pathophysiology and to discover biomarker(s). Therefore, CardioGenBase contains both gene and protein expression profiles of more than 30 and 10 tissues, respectively. In addition, protein-protein interaction (PPI) networks and pathways are provided to understand disease molecular mechanism. Here, the PPI network shows the interaction of disease gene with other key molecules to execute a molecular function(s) through single/multiple pathways[10]. Further, all the associated pathways were given to show the involvement of the query gene in various molecular processes. Furthermore, user can magnify these pathway images in a new window for better perceptive, and those images can be downloaded. Also, the database consists of gene-drug information such as inhibitor, stimulator and suppressor which are helpful in pharmacological studies. All these data are organized into four different tools in the web interface.

### Tool 1: Disease Finder

**Fig 2:**
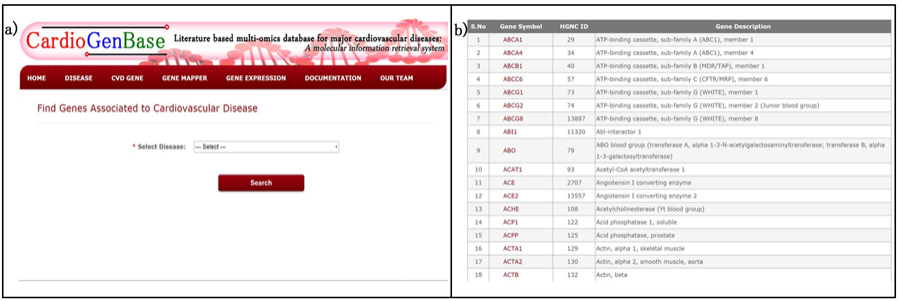
Disease Finder. a) All the reported genes associated a major cardiovascular disease could be retrieved using this query page. b) The result page showing all the genes associated with a disease of interest.

The *disease finder* provides genes that are associated to a major cardiovascular disease (Figure A in S1 File). User can select any cardiovascular disease of their interest from the list to retrieve complete genes of the selected cardiovascular disease. This tool enables the user to identify the reported genes for the given disease condition (Fig 2).

### Tool 2: CVD Gene Finder

**Fig 3:**
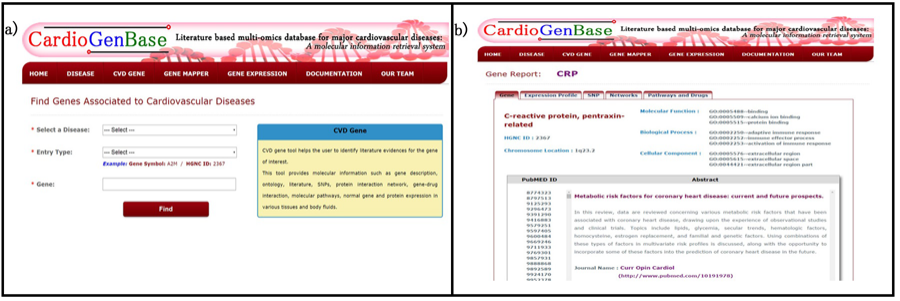
CVD Gene Finder. a) The literature evidence and molecular information could be obtained for a gene of interest. User can search the gene by HGNC ID or gene symbol. b) The output shows the molecular information on the query gene.

*CVD gene finder* allows the user to search for a gene to any major cardiovascular disease covered in the database (Figure B in S1 File). This tool aids the user to search earlier scientific reports on the query gene for the disease of interest (Fig 3). User needs to select an MCVD and the query gene (HGNC ID or official Gene Symbol). The results for the queried gene consists of literature evidences including abstract, Pubmed IDs and journal citation along with the detailed molecular information about the gene such as ontology, SNP, PPI network, pathways, drugs along with the literature evidences.

### Tool 3: Gene Mapper

**Fig 4:**
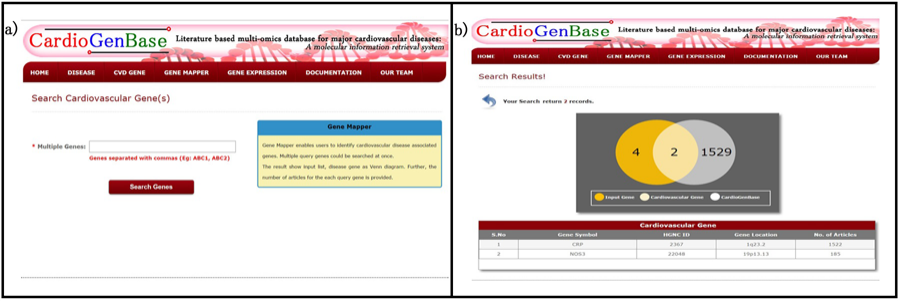
Gene Mapper. a) Multiple query genes can be searched at once. b) The result shows input list, disease gene as Venn diagram. Also, the number of articles for each query gene is provided.

*Gene Mapper* helps the user to search multiple genes at once to identify its cardiovascular disease associated (Figure C in S1 File). The gene Mapper generates a Venn diagram that displays user input gene list and number of cardiovascular associated genes from the input list (Fig 4). For each cardiovascular associated gene, the literature evidence was provided that enable the user to rank or prioritize the query genes based the given literature evidence.

### Tool 4: Gene Expression Finder

**Fig 5:**
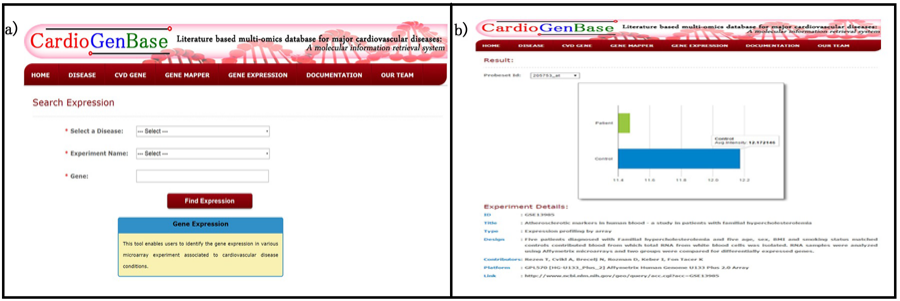
Gene Expression Finder. a) This tool enables users to identify gene expression in various microarray experiments associated to cardiovascular disease condition. b) the result represented as a bar diagram where the raw intensities of grouped samples are given as interactive charts.

*Gene expression finder* enables users to identify the expression of a gene under various cardiovascular disease conditions (Figure D in S1 File) The microarray gene expression data for cardiovascular disease were used retrieved from NCBI GEOSET. Here, the raw intensity of the samples are collected, grouped and the average intensities is displayed (Fig 5). This feature is similar to the NCBI GEO profile viewer[11], but specific to cardiovascular disease conditions. This tool enables the user to identify the differentially expressed genes in the selected experimental condition.

### Comparison and Validation

To our knowledge, CardioGenBase is the only database that integrates six major cardiovascular conditions with gene to publication associations from ~24000 research articles. In order to evaluate the accuracy and credibility of CardioGenBase, the manually curated CADgene database[12] was used as a "gold standard" which was updated in the year 2013. For the fair comparison, the articles published between the years 1988 to2013 was used for the validation process. Three volunteers were assigned to collect fifty test genes associated to coronary artery disease from the articles published between the year 1988 with 2013 (Table 2). The collected genes were tested in both the databases, and their performance was validated by the volunteers. Briefly, out of fifty genes searched, most of them were present in CardioGenBase whereas only thirty six were found in CADgene database. For example, well reported coronary artery disease genes such as ALB[13], HLA[14], IL-2[15], IL-3[16], IL-27[17] and IL-33[18] were not represented with literature evidence in the CADgene database. As a result, the CardioGenBase showed better performance with respect its precision, recall, accuracy and F-measure compared to CADgene database. In addition to the performance, the volume of articles covered in CADgene is about 5000 whereas CardioGenbase contains 8319 for coronary heart disease alone. Importantly, the CardioGenbase includes literature evidence for six major cardiovascular conditions, but CADgene database is restricted only to coronary heart disease. Further, CardioGenBase provides bioavailability, gene and protein expression to aid biomarker discovery. Overall, the CardioGenBase contains more cardiovascular genes than existing databases such as CaGE[19], Phenopedia and Genopedia[20].

**Table 2:** List of fifty genes selected by the volunteers for validation. These fifty genes were searched in CardioGenBase and CADgene database for effective comparison. The result shows that most of the cardiac genes are found in CardioGenBase than CADgene database.

Gene Symbol	Cardiogenbase	CADgene	Volunteers
***ACE***	+	+	Yes
***AKT1***	+	-	Yes
***ALB***	+	-	Yes
***APOC4***	+	+	Yes
***BCL2***	+	+	Yes
***BMP4***	+	-	Yes
***BRCA1***	+	+	Yes
***CASQ2***	+	+	Yes
***CASR***	+	+	Yes
***CBS***	+	+	Yes
***CCL11***	+	+	Yes
***CCL2***	+	+	Yes
***CMA1***	+	+	Yes
***CNDP1***	+	+	Yes
***CREG1***	+	+	Yes
***CRP***	+	+	Yes
***CSF3***	+	-	Yes
***CST3***	+	+	Yes
***EDN1***	+	+	Yes
***EGFR***	+	+	Yes
***EGR1***	+	+	Yes
***ENPP1***	+	+	Yes
***FGA***	+	+	Yes
***HFE***	+	+	Yes
***HGF***	+	+	Yes
***HLA-A***	+	-	Yes
***HLA-C***	+	-	Yes
***HSPB1***	+	+	Yes
***ICAM2***	+	-	Yes
***IL2***	+	-	Yes
***IL27***	+	-	Yes
***IL3***	+	-	Yes
***IL33***	+	-	Yes
***IL5***	+	+	Yes
***IL6***	+	+	Yes
***IL6R***	+	+	Yes
***LCN2***	+	-	Yes
***LDLR***	+	+	Yes
***LPL***	+	+	Yes
***MMP8***	+	+	Yes
***MMP9***	+	+	Yes
***NOS3***	+	+	Yes
***OCA2***	-	-	No
***SLC22A6***	-	-	Yes
***THBS4***	+	+	Yes
***TIMP1***	+	+	Yes
***USF1***	+	+	Yes
***VCAM***	+	+	Yes
***VEGFA***	+	+	Yes
***XRCC3***	+	+	Yes

+ and—symbol indicates presence and absence, respectively. Yes andNo indicates the cardiovascular association.

### Conclusion and future perspectives

CardioGenBase was constructed to provide a comprehensive view of molecular information for the major cardiovascular diseases. It encompasses a broader spectrum of data by integrating the information from both literature and biological databases. In comparison with existing databases, CardioGenBase was created by semi-automated curation of published articles to accomplish the growing demands in the field of cardiovascular research. By providing effective search and browsing features, it operates as a flexible and user friendly platform for the molecular study of MCVDs. In the next few years, the scope of CardioGenBase will be extended to integrate new data sets with systematic updates. We hope our constant efforts would aid in understanding the molecular aspects of MCVDs that would support the global cardiovascular health.

## Materials and Methods

The CardioGenBase provides extensive molecular information for the major cardiovascular diseases. The database was constructed based on (1) literature collection and curation (2) data enrichment (3) system implementation and visualization. Each of these phases is explained in the following sections.

### Literature collection and curation

Gene-to-literature associations in the CardioGenBase were extracted by applying text mining approach on the records available at MEDLINE publications. In general, our approach seeks appearances of disease terms in titles, abstracts and PMC open access full text articles. Highly relevant articles were filtered and subjected to dictionary based text mining approach to extract gene/proteins. The dictionary contains both symbols as well as gene description from human gene nomenclature committee. Lucene was used to process the articles to identify gene/protein names using curated dictionary. Further, the extracted data was manually verified before data enrichment.

### Data enrichment

Besides the identification of disease associated genes from the data mining, it is essential to understand their function at the molecular level. Hence, we have presented several annotations, including molecular function, biological process, cellular component, drugs, pathways, PPIs, gene and protein expression in various tissues and body fluids. Also, the bioavailability of disease-gene encoding protein is given to facilitate biomarker discovery for feasible diagnosis. All the annotation data sets were retrieved from DAVID [21],PANTHER[22], Reactome[23], HPRD[24], NCBI GEO[25], MOPED[26] and OMIM[27]. In addition, the expression profiles of these genes in various microarray datasets were provided to demonstrate their differential behavior in various cardiovascular conditions. The detail usage of the tools in database is provided in Figures A-D in S1 File.

### Cross validation

In order to validate the efficiency of our database, the CardioGenBase was compared with manually curated CADgene database. For reliable comparison, three volunteers were together assigned to collect fifty test genes from the research and review articles published between the years 1988 to 2013(Table 3). Further, the collected test genes were used as query to search in both the databases to determine its precision, recall, accuracy and F-measure.

$$Precision\;=\frac{truepositive}{truepositive+falsepositive}$$

$$Recall\;=\frac{true\;positive}{true\;positive+false\;negative}$$

$$Accuracy=\frac{true\;positive+true\;negative}{true\;positive+true\;negative+false\;positive+false\;negative}$$

$$F-measure=2*\frac{precision\;*\;recall}{precision+recall}$$

**Table 3:** The parameters used validate the database. Statistics were employed to find out the precision, recall, accuracy and F-measure of CardioGenBase. Overall, the results support the viability and quality of data represented in the database.

Parameter	Cardiogenbase	CADgene
**Precision**	100	100
**Recall**	97.95	97.29
**Accuracy**	96.04	72.05
**F-measure**	98.96	98.63

### System implementation and visualization

A user-friendly web interface for browsing was implemented by HTML, CSS, PHP and jQuery. The data sets were stored and managed in MySQL, a popular open source database management system. All the data sets such as abstracts, ontology, gene expression, protein expression, bioavailability, pathways and drugs were maintained as separate tables. Google charts were embedded in the web page for the diagrammatic representation. In addition, jQuery, the cross-platform java script library was designed to simplify client-side scripting of HTML.

## Supporting Information

S1 File CardioGenBase tutorial for user. Describes the procedures and utility of the tools in the database.Disease Finderprovides all the genes reported for a major cardiovascular disease of interest (Figure A). CVD GENE Finderhelps the user to identify literature evidences for the gene of interest (Figure B).Gene Mapper enables users to identify cardiovascular disease associated genes. Multiple query genes could be searched at once (Figure C). Gene Expression Finder enables users to identify the gene expression in various microarray experiment associated to cardiovascular disease conditions (Figure D).
(PDF)
