# Supplementary material for: CardioGenBase: A Literature Based Multi-Omics Database for Major Cardiovascular Diseases
Source: PLoS One. 2015 Dec 1;10(12):e0143188. doi: 10.1371/journal.pone.0143188 (PMC4666633; doi:10.1371/journal.pone.0143188)
Supplement: S1 File — Describes the procedures and utility of the tools in the database.Disease Finderprovides all the genes reported for a major cardiovascular disease of interest (Figure A). CVD GENE Finderhelps the user to identify literature evidences for the gene of interest (Figure B).Gene Mapper enables users to identify cardiovascular disease associated genes. Multiple query genes could be searched at once (Figure C). Gene Expression Finder enables users to identify the gene expression in various microarray experiment associated to cardiovascular disease conditions (Figure D). (PDF) [file pone.0143188.s001.pdf]

CardioGenBase consists of four major tools to fit the user needs. The tutorial for each of these tools are given below.

<u>Figure A Disease Finder</u>

This tool provides all the genes reported for a major cardiovascular disease of interest.

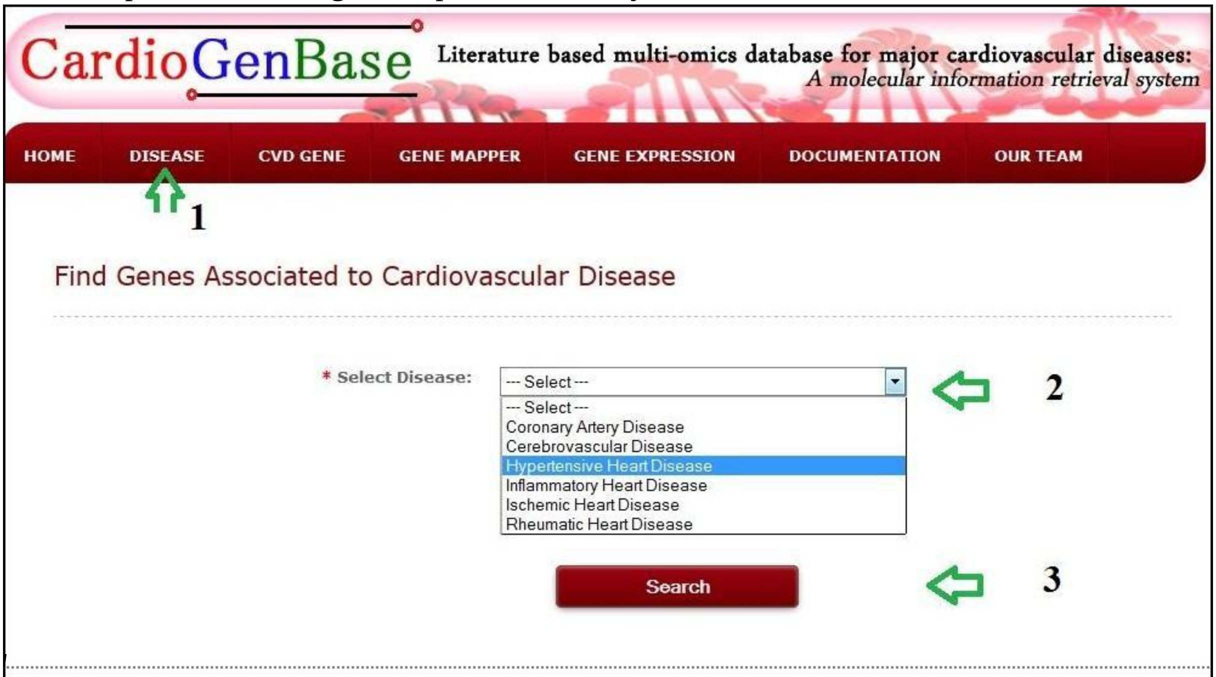

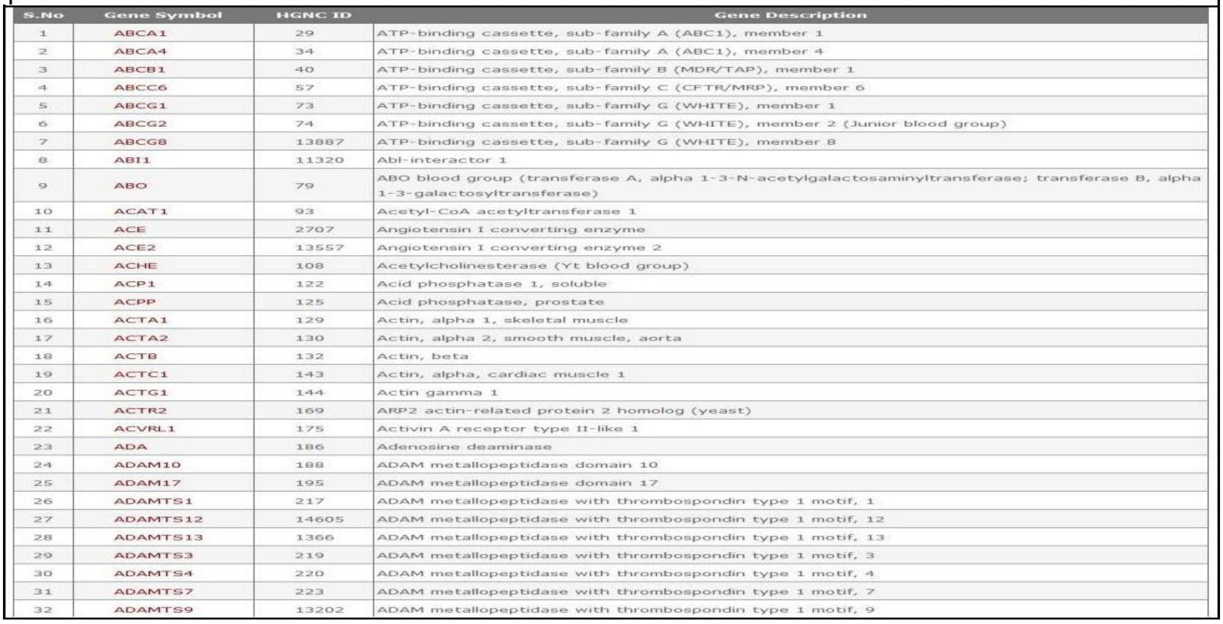

S.No	Gene Symbol	HGNC ID	Gene Description
1	ABCA1	29	ATP-binding cassette, sub-family A (ABC1), member 1
2	ABCA4	34	ATP-binding cassette, sub-family A (ABC1), member 4
3	ABCB1	40	ATP-binding cassette, sub-family B (MDR/TAP), member 1
4	ABCC6	57	ATP-binding cassette, sub-family C (CFTR/MRP), member 6
5	ABCG1	73	ATP-binding cassette, sub-family G (WHITE), member 1
6	ABCG2	74	ATP-binding cassette, sub-family G (WHITE), member 2 (Junior blood group)
7	ABCG8	13887	ATP-binding cassette, sub-family G (WHITE), member 8
8	ABI1	11320	Abl-interactor 1
9	ABO	79	ABO blood group (transferase A, alpha 1-3-N-acetylgalactosaminyltransferase; transferase B, alpha 1-3-galactosyltransferase)
10	ACAT1	93	Acetyl-CoA acetyltransferase 1
11	ACE	2707	Angiotensin I converting enzyme
12	ACE2	13557	Angiotensin I converting enzyme 2
13	ACHE	108	Acetylcholinesterase (Yt blood group)
14	ACP1	122	Acid phosphatase 1, soluble
15	ACPP	125	Acid phosphatase, prostate
16	ACTA1	129	Actin, alpha 1, skeletal muscle
17	ACTA2	130	Actin, alpha 2, smooth muscle, aorta
18	ACTB	132	Actin, beta
19	ACTC1	143	Actin, alpha, cardiac muscle 1
20	ACTG1	144	Actin gamma 1
21	ACTR2	169	ARP2 actin-related protein 2 homolog (yeast)
22	ACVRL1	175	Activin A receptor type II-like 1
23	ADA	186	Adenosine deaminase
24	ADAM10	188	ADAM metallopeptidase domain 10
25	ADAM17	195	ADAM metallopeptidase domain 17
26	ADAMTS1	217	ADAM metallopeptidase with thrombospondin type 1 motif, 1
27	ADAMTS12	14605	ADAM metallopeptidase with thrombospondin type 1 motif, 12
28	ADAMTS13	1366	ADAM metallopeptidase with thrombospondin type 1 motif, 13
29	ADAMTS3	219	ADAM metallopeptidase with thrombospondin type 1 motif, 3
30	ADAMTS4	220	ADAM metallopeptidase with thrombospondin type 1 motif, 4
31	ADAMTS7	223	ADAM metallopeptidase with thrombospondin type 1 motif, 7
32	ADAMTS9	13202	ADAM metallopeptidase with thrombospondin type 1 motif, 9

1) Click **Disease** tab
2) Select disease of interest
3) click **Search**
4) In results, click the gene symbol for more details

Figure B CVD GENE Finder

CVD gene helps the user to identify literature evidences for the gene of interest.

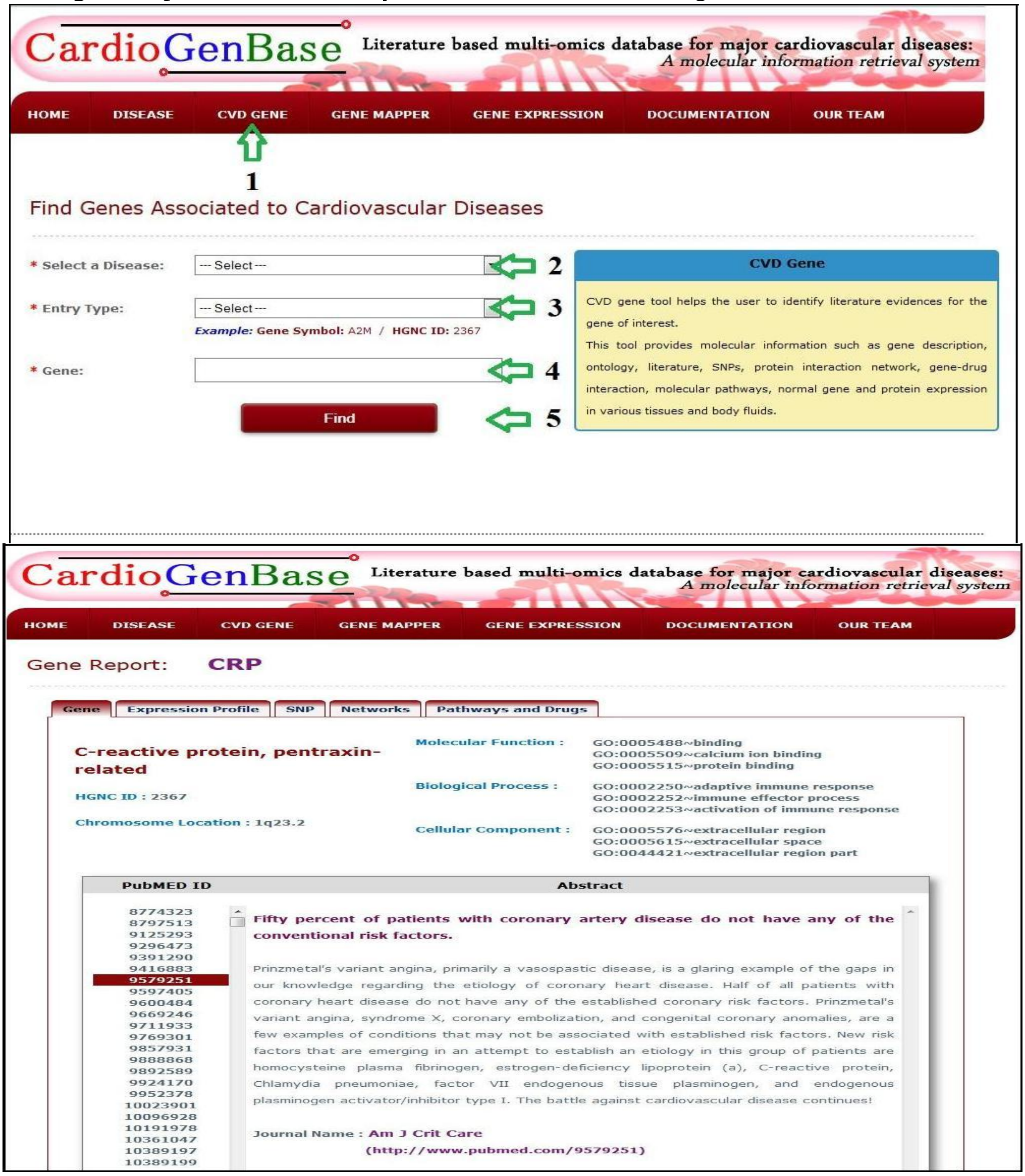

1) Click **CVD GENE** tab
2) Select disease of interest
3) Choose entry type, Gene symbol or HGNC ID
4) Enter the gene
5) Click **Find** to retrieve result

The result includes molecular information such as gene description, ontology, literature, SNPs, protein interaction network, gene-drug interaction, molecular pathways, normal gene and protein expression in various tissues and body fluids.

Figure C Gene Mapper

Gene Mapper enables users to identify cardiovascular disease associated genes. Multiple query genes could be searched at once.

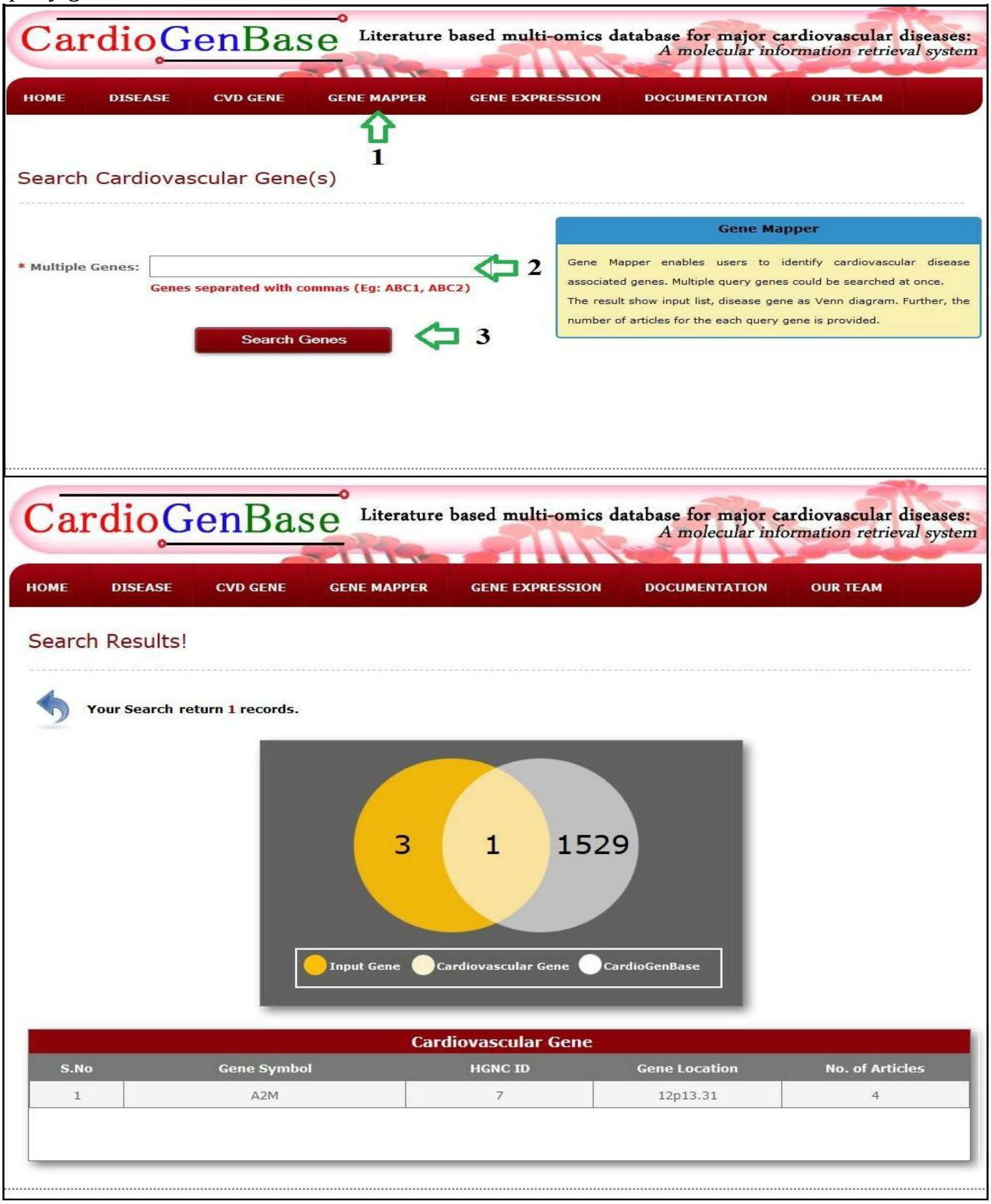

1) click **Gene Mapper** Tab
2) Enter multiple genes separated by comma
3) Click **Search Genes** for results

The result show input list, disease gene as Venn diagram. Further, the number of articles for the each query gene is provided.

Figure D Gene Expression Finder

This tool enables users to identify the gene expression in various microarray experiment associated to cardiovascular disease conditions.

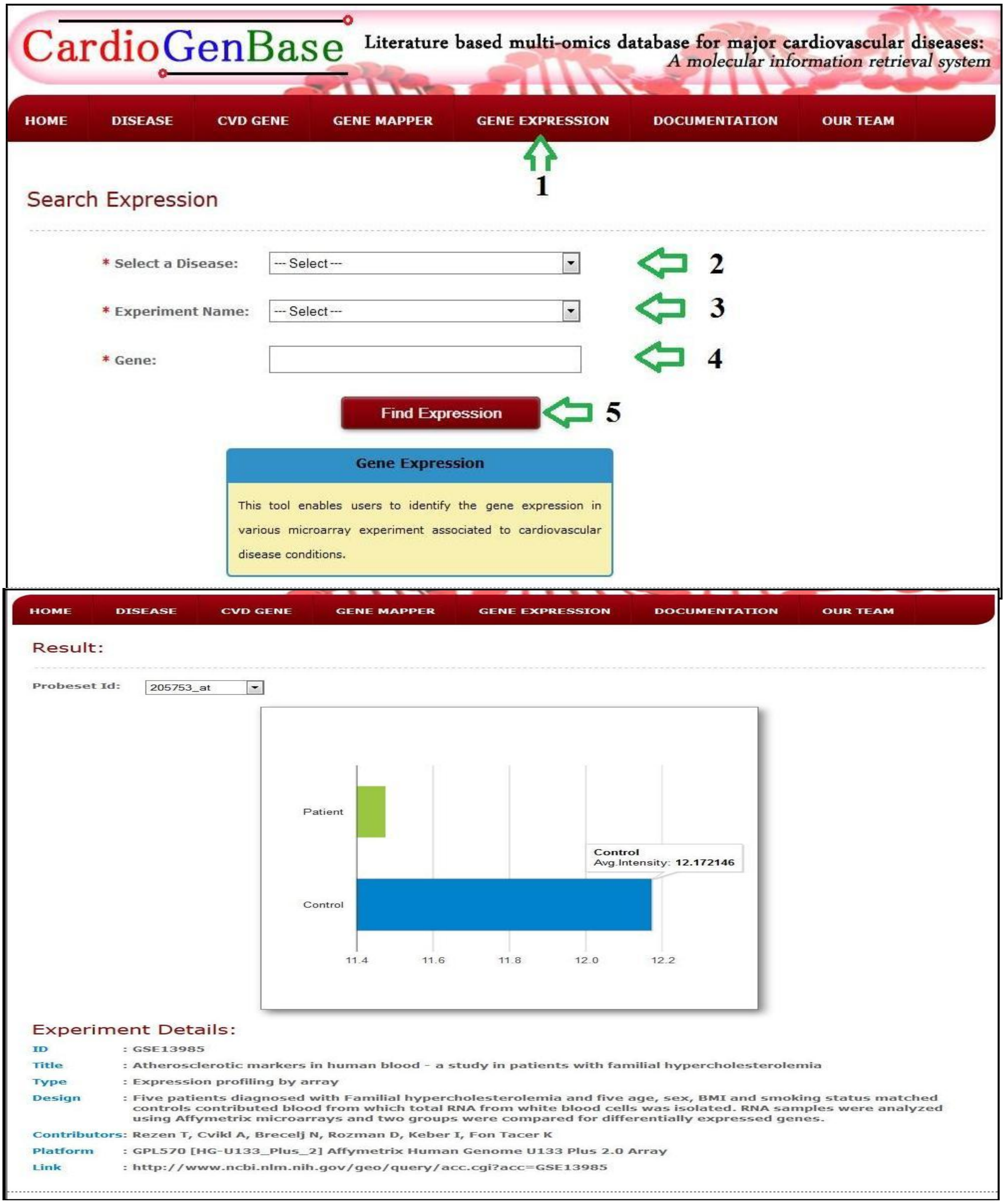

1) Click **Gene Expression** tab
2) Choose a disease
3) Select a experiment of interest
4) Enter a gene symbol
5) Click **Find Expression**
